# Evaluating Generative AI for Deprescribing: Accuracy, Safety, and Clinical Utility

**DOI:** 10.1093/geroni/igaf122.1185

**Published:** 2025-12-31

**Authors:** Juliessa Pavon, Cara McDermott, Marc Pepin, William Bryan, Ivuoma Igwe, Cathleen Colon-Emeric

**Affiliations:** Durham VA Medical Center, Durham, North Carolina, United States; Duke University, Durham, North Carolina, United States; Durham VA Health Care System, Durham, North Carolina, United States; Durham VA Health Care System, Geriatric Research Education and Clinical Center (GRECC), Durham, North Carolina, United States; Durham VA Health Care System, Geriatric Research Education and Clinical Center (GRECC), Durham, North Carolina, United States; Duke University School of Medicine, Durham, North Carolina, United States

## Abstract

Limited geriatrics and pharmacy resources in VA Medical Centers (VAMCs) necessitate innovative strategies to enhance deprescribing efforts. Generative AI platforms, such as OpenAI, have the potential to generate deprescribing recommendations, tapering schedules, and patient education materials by synthesizing information from medical literature and drug interaction databases. When integrated into deprescribing programs, these platforms could enhance scalability and sustainability by providing real-time, context-aware decision support. However, before implementation, it is essential to assess their safety, accuracy, and potential risks, including errors, omissions, and confabulations. Using the VA LLM platform TryOpen AI 3.5, this project assesses AI-generated deprescribing recommendations compared to those generated by an interprofessional team of pharmacists, geriatricians, and nurses, using de-identified case scenarios (N = 100) from our VA deprescribing program. We assess AI performance through content analysis, identifying recurring themes (e.g., medication selection, tapering regimens, side effects, and patient education) using the HELM criteria (Holistic Evaluation of Language Models), which assesses accuracy, uncertainty, efficiency, fairness, and bias. Findings will inform safe AI integration in deprescribing programs, identifying appropriate applications and potential safety risks to enhance medication management for older Veterans.

